# Principles of care for pregnant and parenting people with substance use disorder: the obstetrician gynecologist perspective

**DOI:** 10.3389/fped.2023.1045745

**Published:** 2023-05-24

**Authors:** Cecily May Barber, Mishka Terplan

**Affiliations:** ^1^ Grayken Center for Addiction, Boston Medical Center, Boston, MA, United States; ^2^ Department of Obstetrics and Gynecology, Boston University School of Medicine and Boston Medical Center, Boston, MA, United States; ^3^ Friends Research Institute, Baltimore, MD, United States; ^4^ Department of Family and Community Medicine, University of California, San Francisco, CA, United States

**Keywords:** addiction, pregnancy, parenting, disparities, stigma

## Abstract

Substance use in pregnant and parenting persons is common, yet still underdiagnosed. Substance use disorder (SUD) is one of the most stigmatized and undertreated chronic medical conditions, and this is exacerbated in the perinatal period. Many providers are not sufficiently trained in screening or treatment for substance use, so gaps in care for this population persist. Punitive policies towards substance use in pregnancy have proliferated, lead to decreased prenatal care, do not improve birth outcomes, and disproportionately impact Black, Indigenous, and other families of color. We discuss the importance of understanding the unique barriers of pregnancy-capable persons and drug overdose as one of the leading causes of maternal death in the United States. We highlight the principles of care from the obstetrician-gynecologist perspective including care for the dyad, person-centered language, and current medical terminology. We then review treatment of the most common substances, discuss SUD during the birthing hospitalization, and highlight the high risk of mortality in the postpartum period.

## Introduction

Substance use in pregnant and parenting persons is common, yet both underdiagnosed and undertreated in part due to misunderstanding and misinformation regarding substance use, misuse, and use disorder in pregnancy (1).

Most people in the US use drugs (opioids, alcohol, nicotine/tobacco, stimulants, and cannabis) to which some people develop an addiction, including people who are capable of pregnancy (2). Most people quit or cut back substance use when they become pregnant (3, 4). However, those who continue to use, likely have a substance use disorder (SUD) (5). Addiction, or SUD, is a chronic and treatable medical condition (6). Untreated SUD is associated with preterm delivery, low birth weight, and other negative birth outcomes. In contrast, people with treated addiction are more likely to deliver a normal weight infant at term (7). The old adage “healthy mother equals healthy baby” applies to addiction as it does to other chronic diseases in pregnancy.

Universal assessment of behavioral health is recommended in prenatal care (8–14); however, it is unevenly actualized and some providers and health systems deploy drug testing in place of proper screening (15, 16). Although the effectiveness of treatment in pregnancy is well established, most pregnant people receive no addiction treatment and treatment (including medication) is inequitably distributed across populations (17, 18). In short, the field of addiction medicine as it concerns pregnancy and parenting suffers not from gaps in scientific knowledge, as much as it suffers from gaps in implementation. We know how to care for pregnant and parenting people with SUD. Many of the principles of care were first described almost 50 years ago (19, 20).

Pregnancy and postpartum are a period of significant change both biologically and socially and can lead to new or increased contact with healthcare providers. In this role, all providers have an obligation to present information, resources and support that strengthen this family unit. The complexity of the social and historical context in which people who use substances interact with the healthcare system is highlighted during the perinatal period (21)—most notably, this population faces unique threats of legal repercussions for reporting use and seeking treatment (22). This threat and stigma can lead to significant trauma for pregnant and postpartum persons and disproportionately affects non-White families (23).

This manuscript reviews the principles of care of pregnant and parenting people with SUD from the obstetrician gynecologist perspective. The authors are both obstetrician gynecologists and addiction medicine providers with combined almost three decades of clinical experience. The discussion is centered on the dyad—on the indivisible connection between a pregnant person and the fetus (the “maternal-fetal unit”) and, following birth, the connection between a new mother and an infant—and grounded in both foundational texts as well as contemporary data.

## Principles and context

Health care should be both evidence-based and person-centered. Pregnant people with SUD experience discrimination, are denied dignity and respect, and often lack access to evidence-based care (24). Pregnant people with SUD often have a significant history of trauma, including childhood physical or sexual abuse and current intimate partner violence (25–27). Many have interacted with the child welfare system in the past and the potential of child welfare involvement postpartum looms over the entire perinatal period (28, 29). Finally, the birth experience can be traumatizing which can reaffirm existing medical mistrust (30).

To address discrimination and structural inequities, to reflect evidence-based practice, and to actualize person-centered care, attention should focus first on language.

Language is important. The words we use can convey (intentionally or unintentionally) stigma and prejudice (31, 32). Creating a non-judgmental environment is important to provide appropriate care for persons with substance use disorder. Therefore, providers need to model language that is both evidence-based (i.e., clinically accurate) and person-centered (i.e., non-stigmatizing). Providers should avoid slang and use language that clearly communicates that substance use disorder is a chronic medical condition, that emphasizes treatment (especially medication and behavioral health), and that promotes recovery (see Table 1) (31, 33, 34). Finally, it is important to note that “stigma catches up” and therefore language that reflects the dignity and humanity of pregnant and parenting people who use drugs is constantly changing. As healthcare professionals, it will be necessary to adapt our terminology as needed.

**Table 1:** Addressing stigma by changing the language we use.

Stigmatizing language	Preferred language
Addict, junkie, abuser	Person in active addiction Person with a substance use disorder Person in recovery (1)
Addicted baby	Neonate with in-utero exposure to [substance] Neonatal abstinence syndrome (2)
Substance abuse	Substance use or misuse Substance use disorder
Clean or sober	Abstinent SUD in remission Testing “negative” for [substance]
Dirty	Using [substance] Testing “positive” for [substance] (3)
Replacement or substitution therapy, medication-assisted treatment	Medication for opioid use disorder (MOUD) Treatment
Getting or being high	Intoxicated Under the influence of [substance]
Shooting up	Intravenous or injection drug use
Relapse	Return to use SUD recurrence

## Screening and assessment

Substance use and use disorder are common—nearly 1 in 5 pregnant women report any substance (including tobacco, alcohol, or illicit substances) within the past month, and rates of opioids, cannabis, and stimulants in pregnancy have increased in the past decade (2).

Universal verbal screening for substance use and misuse using a validated instrument is recommended as a routine part of prenatal care by professional societies and public health agencies (8–11, 35, 36). In addition, participation in screening is considered voluntary and rests upon the bioethical principle of autonomy and opposition to coercion (see Table 2) (36–38). Hence, it is recommended that providers ask permission prior to screening and recognize the patient's right of refusal if screening is declined. However, universal screening is vastly underutilized and “risk based” screening persists—a practice that operationalizes and perpetuates stigma and with no improvement of diagnostic accuracy (15, 39).

**Table 2:** Tips for addressing mistrust and making clinical care welcoming.

Permission for Screening	Is it ok if I ask you some questions about smoking, drinking and other drugs?
Prenatal Care and SUD Treatment	What are you looking for in a provider? What is the most important thing to you about treatment or recovery?
Medication Decisions	What do you know about [methadone or buprenorphine]? Do you have any concerns from prior treatment experiences?
Postpartum Care	What do I need to know about your birthing experience to help you create an environment to best care for you and your child? What do you need to care for your infant?
In General	What do you need to feel safe?

There are multiple validated tools to identify problematic substance use including maternal interview and screening questionnaires. Although many screening instruments have been used in pregnancy, only two studies have directly compared the screening instrument performance in pregnancy. Ondersma et al compared 5 instruments: the Substance Use Risk Profile—Pregnancy (SURP-P), CRAFFT (acronym for five-item screener with items related to car, relax, alone, forget, friends and trouble), 5Ps (parents, peers, partner, pregnancy, past), Wayne Indirect Drug Use Screener (WIDUS), and the National Institute on Drug Abuse (NIDA) Quick Screen (40). Coleman-Cowger et al compared 3 instruments: 4P's Plus, NIDA Quick Screen-ASSIST, and the SURP-P (41). No instrument was superior in any of the analyses. Therefore, it is recommended that providers use whatever validated tool is most able to be integrated into the electronic health record.

Drug **screening** captures behaviors related to substance use. In contrast, drug **testing** is the evaluation of a biological matrix (such as urine, hair, or meconium) for the presence of drug metabolites or parent compounds. Drug testing is not recommended as an appropriate means to identify substance use or misuse, much less addiction (8, 36). The information obtained from a drug test is not uniform as the time of detection varies greatly across substances. In addition, both false positive and false negative results are common in drug tests (42, 43). Furthermore, the American Society of Addiction Medicine (ASAM) recommendations are clear that definitive testing (i.e., gas chromatography) is required “when the results will inform a decision with major clinical or non-clinical implications for the patient” which, given the realities of child welfare reporting, would be any drug test in pregnancy especially during the birthing hospitalization (44).

Screening is not diagnosis, and a positive screen must be followed up with the assessment of DSM criteria for the establishment of diagnosis. Screening helps to stratify patients into risk categories: those that meet criteria for a use disorder need treatment; those with “moderate risk” (history of use but without addiction) should receive a brief intervention grounded in motivational interviewing and (more) frequent follow up visits; and those with low risk (no past or current use) should receive brief advice and acknowledgement of their healthy behaviors (11).

Although screening is recommended, patients may be legitimately fearful of disclosure, including the threat of legal repercussions (45). It is important that all providers continue to create a safe and non-judgmental environment that encourages engagement in care. Providers should be honest and transparent regarding how information obtained from either screening or testing is shared with external agencies, including child welfare, and overreporting (use or positive drug testing without protective concerns) should be discouraged.

## Treatment

SUD is a chronic medical condition and like other chronic conditions, outcomes are greatly improved by treatment (46). There is, however, a large gap in access to treatment (both medication and behavioral) by substance type, with the highest rate of treatment for opioids and the lowest for alcohol (17). The marked gap in treatment is further magnified by racial inequities. Compared to White individuals, Black and Hispanic populations are less likely to receive any SUD treatment, less likely to receive medication for OUD (MOUD), and if in treatment, receive lower doses of MOUD (18, 47).

Among people with SUD, polysubstance use is common and co-occurring substance use and use disorders can be treated simultaneously. Ideally, care should be delivered in a comprehensive and co-located capacity, that is, through the integration of addiction treatment and prenatal care. Integrated care models have been described since the 1970s, are considered the “standard of care”, and are associated with improved birth outcomes (7, 20). However, some individuals need a higher level of care. ASAM categorizes addiction treatment along levels of care that range from outpatient to residential and inpatient (48). Level of care should be evaluated at treatment intake and throughout, especially if treatment is failing a patient at a particular moment. Medication should be available throughout levels of care as should childcare services, although both medication and childcare are often absent and are hence barriers to care for pregnant and parenting people (49, 50). In addition, treatment should address concomitant mental health disorders (51). Below we discuss treatment and outcomes by substance type.

## Opioids

From 1999 to 2014, the rate of OUD complicating birth has increased by more than 4-fold and in some states the rate has increased nearly 10-fold, yet OUD treatment is still stigmatized and underutilized (8, 52). Despite public health and professional society recommendations supporting MOUD (14), pregnant and postpartum individuals with OUD continue to face barriers to treatment, including stigma (53), discrimination, lack of knowledgeable clinicians (54), and misinformation about NAS (55). Available data regarding negative fetal effects of opioids are inconsistent and some of the literature is cross-sectional, retrospective (hence subject to recall and other bias), or outcome assessments are not masked. However, there is no evidence that MOUD (either buprenorphine or methadone) increases risk of congenital anomalies (56).

MOUD, including methadone (a full mu-agonist and weak NMDA receptor antagonist), buprenorphine (partial mu-agonist with a high affinity for the mu-opioid receptor and partial mu-antagonist) and naltrexone (nonselective opioid receptor antagonist), save lives and are the standard OUD treatment—among pregnant and postpartum individuals (8, 36). MOUD improve both substance use and pregnancy outcomes and decrease overdose risk compared with no treatment (57). Evidence for the safety and efficacy of methadone and buprenorphine is the strongest, however due to recent studies confirming safety and efficacy of the buprenorphine-naloxone combination product and persistent barriers to outpatient methadone access in the current flawed system, buprenorphine use is increasing (58), and a multisite injectable buprenorphine trial in pregnancy is ongoing (59). Detoxification, or medically supervised withdrawal, is not recommended in pregnancy. Naltrexone is the newest approved MOUD and the least studied in pregnancy; the urgency to close this research gap is currently being addressed in an ongoing multisite trial (59). Detoxification is not treatment, is associated with return to use and not associated with a decrease in NAS (60). However, it is important to respect patient autonomy and it may be attempted after shared decision making with counseling on the safety of detoxification and the risk of return to use (61).

Although MOUD with either methadone or buprenorphine are the safest and most effective treatment for OUD in pregnancy, most people with OUD receive no treatment in pregnancy (17), only 50% of people admitted to specialty addiction treatment programs receive medication (62), and there are marked racial inequities in medication receipt, type, and dose. Black pregnant people are less likely to receive medication compared to White pregnant people and overall, more likely to receive methadone than buprenorphine (18). Even among those who receive methadone, mean daily doses are highest for White (144.9 mg) compared to Black (97.5 mg) pregnant people (47).

MOUD dose (methadone or buprenorphine) is not correlated with neonatal abstinence syndrome (63, 64), however there is decreased incidence and severity of NAS associated with buprenorphine compared to methadone (65). Behavioral interventions, such as contingency management, cognitive behavioral therapy, and family therapy, are helpful (66, 67) but absence of or non-adherence with behavioral health should not be used as justification to withhold MOUD (68). Optimal duration of treatment with MOUD is not established and for some individuals may be lifelong, however MOUD discontinuation postpartum is common and is associated with increased rates of return to use, overdose, and death (57, 69). Providers should be aware of community resources including peer recovery support services and “12 step programs” because use of peer services is associated with increased attendance at OUD medical appointments (70). Recent qualitative studies suggest that peer services are valued among pregnant and postpartum individuals with OUD and are increasingly accessible through telehealth and online (71).

Naloxone (short-acting opioid antagonist) rapidly reverses opioid overdose and is not considered MOUD. Given the increase in intentional and unintentional fentanyl use, pregnant and postpartum individuals and their supports should be counseled on the increased risk of overdose, need for immediate naloxone administration in case of overdose, and to call emergency medical services because multiple doses of naloxone and/or oxygen support may be needed (72). Because fentanyl is increasing throughout the US and there are increasing reports of xylazine (alpha-2 adrenergic agonist) in the drug supply, further training in overdose prevention and management is needed. Xylazine is sympatholytic causing severe sedation, respiratory depression and slowed heart rate, further complicating overdose prevention and management, therefore provider training is needed on co-prescription of naloxone, education on naloxone-resistant overdoses, and increasing need for respiratory support in xylazine-opioid combination use and patient support training on overdose recognition (73).

## Alcohol

Binge alcohol use—4 or more drinks on a single occasion for women—is common in pregnant individuals in the past month, yet rates of alcohol use and alcohol use disorder (AUD) are likely gravely miscalculated (2). Although alcohol is an established teratogen and there is no known safe lower limit of alcohol exposure. Fetal alcohol syndrome (FAS) is the leading modifiable cause of intellectual disability and developmental delay in the US, with a similar rate to Down syndrome, and 20 times more common in the US (1.95/1,000) than in Europe (0.08/1,000) (74). All individuals who report alcohol use should be evaluated for AUD and referred to treatment to avoid withdrawal which can be life-threatening. Untreated withdrawal is associated with a nearly 5% subsequent yearly mortality rate (75). The American Society of Addiction Medicine recommends initial inpatient management for individuals at risk of severe withdrawal, which includes pregnancy (76). Withdrawal management alone is not addiction treatment—the mainstay of treatment for AUD is medication with behavioral interventions because behavioral interventions alone are associated with high rates of return to use (77, 78). There are three approved medications for AUD: disulfiram (aldehyde dehydrogenase inhibitor), acamprosate (glutamate/GABA neuromodulator) and naltrexone (mu-opioid receptor antagonist). Due to the exclusion of pregnant individuals from medication trials, there is no evidence base regarding safety and effectiveness in pregnancy (79), and these medications are vastly underutilized. However, medications for AUD are almost certainly less harmful than untreated AUD and should be considered in the clinical care for pregnant and postpartum individuals (80, 81).

## Nicotine/tobacco

There is significantly higher tobacco use in individuals who have other SUDs compared to those with no SUD and this does not change dramatically among pregnant and parenting individuals. Although 50% of individuals stop smoking during pregnancy, up to 90% resume within 1 year postpartum (82). Behavioral interventions, such as cognitive behavioral therapy (CBT) or contingency management, remain the standard treatment for smoking cessation (83). Nicotine replacement therapy (NRT) has not been shown to be beneficial in smoking cessation in pregnancy, however, NRT can reduce maternal and fetal exposure to nicotine, mitigate maternal lung disease, and reduce second and third-hand smoke exposure postpartum. Although there are limited data on bupropion and varenicline use in pregnancy, a recent analysis suggests safety of bupropion both in pregnancy and breast/chestfeeding (84). Medications decisions, including NRT, should be individual clinical decisions, balancing risks and benefits, and center on patient autonomy.

## Cocaine

Cocaine use in pregnancy has a shameful history of unscientific and racist rhetoric, filled with false claims of adverse child neurodevelopmental outcomes, teratogenicity, and lifelong mental and physical disability (53, 85–87). It is important to acknowledge that despite the research disproving these claims, significant stigma persists particularly for Black individuals. Because cocaine can cause hypertensive emergencies and increase myocardial oxygen demand, *in utero* exposure can be associated with preterm birth, placental abruption, preeclampsia-like symptoms, maternal coronary artery vasospasm, and myocardial ischemia, infarction or arrhythmia (88). A symptom of both the ongoing “war on drugs” and the subsequent unequal burden of “crack” or crystal cocaine use in Black and poor communities, research on cocaine use disorder (CUD) treatment is limited and there are no medications approved for (CUD) (89). There is modest evidence for bupropion, topiramate, and psychostimulants, but none of these have been studied in pregnancy (89). There is increasing evidence that contingency management increases abstinence, and behavioral modalities remain the mainstay of treatment in pregnancy (67, 90).

## Methamphetamines

Methamphetamine is the second most used illicit substance globally and use has been increasing in pregnancy particularly in Western and rural regions of the US (present in 1% of births) and now also in Eastern and urban regions (particularly via injection and not inhalation) (91). Concurrent opioid and methamphetamine use and methamphetamine-related overdose rates are rapidly increasing globally and this trend is being described as “a new or fourth wave in the opioid crisis” (92). Methamphetamine has vasoconstrictive properties and is associated with preterm birth, low birth weight, and small for gestational age (93), but it not associated with placental abruption. The IDEAL (Infant Development and Lifestyle) is a prospective multisite cohort study designed to prevent repeating the racism and misleading science of early cocaine research (94) Results from IDEAL have demonstrated no differences in child development or motor skills at 3 years of age (95). There are no medications approved for treatment of methamphetamine use disorder (MUD) and none have been studied in pregnancy, however a systematic review of different medications and combinations, demonstrated possible positive effects on treatment outcomes most consistently with stimulant agonists, naltrexone and topiramate (96). There is also some evidence that mirtazapine results in a small reduction in methamphetamine use, less methamphetamine-positive urine tests, and decreased high risk sexual behaviors, yet it does not increase retention in treatment (97). Treatment rests primarily contingency management and motivational interviewing (67, 90).

## Benzodiazepines

Benzodiazepines have benefit in the management of acute seizures and alcohol withdrawal, but they do not improve outcomes in the chronic management of depression or anxiety beyond the first month of treatment (98). Despite this, there are no national guidelines for prescribing and few interventions have been evaluated to reduce benzodiazepine-related problems (99). Yet benzodiazepines are ubiquitous and play a large role in the overdose crisis in the United States because concurrent use with opioids increases the risk of opioid-related accidental poisoning, particularly in the first 90 days of a new prescription (100). Benzodiazepines are prescribed more commonly to women as compared to men and may be over prescribed in pregnant and parenting individuals (101). Benzodiazepines are one of the most frequently prescribed medications in pregnancy: in privately insured individuals, 0.8% have a benzodiazepine prescription (102). Although concurrent use of benzodiazepines and opioids (including MOUD) is associated with overdose and death, MOUD should be continued despite patient report or detection of benzodiazepine use (103). Although *in utero* exposure does not suggest teratogenicity or negative effects on neurocognitive development in children, extended *in utero* exposure can cause neonatal withdrawal symptoms similar to opioids and is associated with longer NAS treatment especially in the context of methadone (104–106).

Similar to alcohol, abrupt cessation of benzodiazepines can be severe and life-threatening. Acute withdrawal is assessed and managed similarly to alcohol withdrawal; however benzodiazepine use disorder (BUD) requires more than withdrawal management and often includes gradual outpatient tapers, which have been shown to have higher efficacy as compared to short-term inpatient care (107). Effective treatment for BUD includes cognitive behavioral therapy and given the similarities with AUD should include consideration of medications for AUD (90).

## Cannabis

Cannabis is the most common substance used in the United States that is illegal under federal law. Approximately 5% of pregnant individuals report past-month cannabis use (2). Many individuals use cannabis to self-treat medical and mental health conditions prior to pregnancy and can continue this use into and during pregnancy especially for pregnancy specific symptoms such as nausea and vomiting (108, 109). Providers should also be aware of the increasing number of synthetic cannabinoids—also known as spice or K2—and of the limited data on maternal and perinatal outcomes (110). Synthetic cannabinoids have more potent effects than natural cannabinoids including respiratory distress, hypertension, acute renal failure, coagulopathy, psychosis, suicidal ideation, and death (111). The American College of Obstetricians and Gynecology, the American Academy of Pediatrics, and the US Surgeon General all advise against medical or recreational cannabis during preconception and pregnancy and lactation due to unknown and potential harmful maternal, fetal and child outcomes (112). Although there are no approved medications for cannabis use disorder, there is some evidence for consideration of *N*-acetylcysteine and gabapentin and there is good evidence for behavioral interventions including motivational enhancements, cognitive behavioral therapy, and contingency management (113).

## The birthing hospitalization

Prior experiences of discrimination in healthcare settings, concern about pain management, and legitimate fear regarding child welfare intervention make the birthing hospitalization a stressful time for pregnant people with SUD.

For patients receiving MOUD, the medication should be continued throughout the birthing hospitalization (8, 36, 90). Dose verification through medical record review, prescription drug monitoring program, or contact with the opioid treatment program is helpful. MOUD does not provide analgesia, should be considered the patient's “baseline”, and people with OUD may require more analgesia compared to people without OUD. Providers discuss options for pain control during prenatal care and upon admission to the birthing hospitalization using a trauma-informed approach founded upon shared decision-making. Some patients may have fears about how opioids analgesia could impact their recovery or may want to avoid opioids altogether, so ascertaining patient preference for pain management is paramount.

All MOUD (including methadone, buprenorphine, and naltrexone) should be continued including perioperatively for scheduled cesarean delivery to decrease the risk for both return to use and a difficult transition back to MOUD after acute pain has resolved (114). Existing research confirms better pain control with protocols that account for increased pain sensitivity and higher opioid tolerance in patients with OUD (115). This approach requires higher doses of short-acting opioids and a multimodal analgesic regimen.

Labor analgesia should be multi-modal and include topical, regional, and systemic approaches. Short-acting opioids can be safely prescribed and co-used with MOUD, including those on injectable naltrexone. Again, discussion of patient preference and a safety plan for opioids at home is crucial. Some patients may want opioids while in the hospital but may not want to have a prescription on discharge, while others may feel safer with a lock box, additional support, or family involvement. All people who may witness an overdose, including people with OUD and those receiving MOUD, should be co-prescribed naloxone upon hospital discharge (116, 117).

The American Academy of Pediatrics state that maternal substance use is “not a categorical contraindication to breastfeeding” (118). Breast or chest feeding is an important nonpharmacologic management of NAS and can be beneficial for all patients in recovery, although sociodemographic characteristics, race, and mental health status are all associated with decreased provider and nursing support of breastfeeding (119). Due to insufficient data on neurodevelopmental outcomes or risk of vertical infection transmission, breastfeeding in the context of continued illicit substance use is not encouraged. Clinical decisions regarding breastfeeding during the birthing hospitalization, however, must rest on the principles of bioethics including respect for bodily autonomy and adequate support to those who choose to breastfeed should be provided (118).

Assessment of behavioral health is an important component of admission and management during the birthing hospitalization. For people who present with untreated substance use disorder, the birthing hospitalization is a critical time to initiate treatment and bridge to continuing care. However, drug testing is grossly overused and misinterpreted despite professional society recommendations against routine drug testing of either the pregnant person or the newborn (120). A positive drug test result is not evidence of health or ill health, is not listed as a criterion for newborn discharge and is not essential to the diagnosis of NAS (121, 122). Yet presumptive positive drug test results are often reflexively reported to child welfare. This practice of “test and report” which reveals the drug test as not clinical but a primarily moral or parenting test, has been criticized by ACOG: “The laws, regulations, and policies that require health care practitioners and human service workers to respond to substance use and substance use disorder in a primarily punitive way, require health care providers to function as agents of law enforcement” (123). Although rare States mandate drug testing during the birthing hospitalization, Federal legislation is clear. CAPTA (the Child Abuse Prevention and Treatment Act) neither requires testing, nor the reporting of positive test results to child welfare and states unequivocally that substance use is not in-and-of-itself an indication of child abuse. Providers rarely understand the consequences of a report (124) and operate under the false assumption that an agency of surveillance can provide necessary services to families (125). Rates of child removal attributed to substance use have doubled in recent years, and the majority of infant reports come from health professionals (126).

As previously discussed, for all patients, it is crucial to prescribe naloxone upon discharge, particularly if co-prescribing opioids, but the need for naloxone should be assessed in all patients with OUD. Additionally, many geographic regions increasingly have a contaminated/poisoned illicit drug supply and therefore, there is an increase in unintentional fentanyl exposure. Safety around the potential for unintentional fentanyl use (i.e., in those with non-opioid SUD) should be addressed and patients with any SUD should be encouraged to have a prescription for or access to naloxone.

## Postpartum

Postpartum is a time of increased vulnerabilities for return to use, SUD recurrence, overdose, and overdose death. Care, which had been focused on the pregnant person, becomes less frequent and shifts from mother to infant and from medical to non-medical domains. Insurance churn, including loss of Medicaid coverage, contributes to care discontinuation especially for addiction treatment (57, 69, 127).

Maternal deaths have been increasing in the US and recent population-based data shows that the peak incidence of self-harm related death (specifically overdose and suicide) is 9–12 months postpartum (128–131). In contrast to a global trend in reduction of pregnant- and postpartum-related deaths, a large observational study reported a 26% increase in maternal deaths in 48 US states from 2000 to 2014 (131). Rates of discontinuation of MOUD have been shown to be greater than 50% at 6 months postpartum. Drug deaths are now the leading cause of maternal death in the US (69, 129). Naloxone prescribing and training is essential at delivery hospitalization discharge as is linkage to continuing care.

High rates of co-occurring mental health and substance use disorders put postpartum persons with SUD at especially high risk of psychiatric morbidity and mortality. Having any substance use disorder or use of illicit substances is associated with at least a threefold greater suicide risk (132). Additionally, many pregnant persons stop treatment, particularly medications, during pregnancy and do not resume immediately postpartum (133). Therefore, postpartum persons with SUD should be screened early and repeatedly for anxiety, depression and IPV in the postpartum period and should continue to follow closely with their addiction, obstetric and behavior health providers. Updated in 2018, ACOG recommends screening for depression, anxiety and IPV in all trimesters and the postpartum period and now recommends more and earlier postpartum follow up visits (134).

Appropriate continuing care should include postpartum services, Hepatitis C virus (HCV) treatment (if applicable), assessment of risk and discussion of initiation of pre-exposure prophylaxis (PrEP) for HIV, family planning counseling, behavioral health, peer recovery support and a “warm handoff” transition to primary care. Providers can partner with community-based organizations, peer support services, home visiting, and Early Head Start to support families and keep people engaged in care. The standard single postpartum visit from the prenatal care provider is likely insufficient for the needs of parenting people with SUD. ACOG recommends redesigning postpartum care to optimize health by focusing on care as an ongoing process with services and support tailored to individual and family needs (135). Postpartum care includes reproductive life planning and the provision of contraception within a shared decision-making context. Having an SUD is associated with higher rates of unintended pregnancy compared to the general population, especially in the immediate postpartum period (136)—86% of persons with SUD did not plan their pregnancy compared to 31%–47% in the general population (137). Contraceptive use, particularly the more effective LARC methods, among persons with SUD is lower (138, 139), however current data suggests that lower uptake of reproductive health services in this population is complex. A recent study found an association between increased postpartum contraceptive use and increased prenatal care visits, OBGYN buprenorphine prescribing and increased postpartum visits (140). Furthermore, HCV infection is rapidly increasing among reproductive-aged persons with injection drug use (141) leading to an ACOG recommendation for universal HCV screening in pregnancy (142). Enhanced engagement in care during pregnancy should not be a missed opportunity to provide persons with OUD access to treatment of other medical disorders such as HCV treatment.

## Conclusion

Although drug use decreases significantly during pregnancy and continues to decrease from the first to the third trimester, overall substance use—including opioids, alcohol, cannabis, and cocaine—is increasing among pregnant people in the US. Substance use trends, treatment access, and child welfare policies differ widely by state and therefore it is important that all providers understand their specific geographic resources and climate. SUD outcomes are significantly improved by treatment, however a large gap in treatment access persists. Among those with need for SUD treatment, only 11% report receiving treatment. Although pregnant people are considered a priority population, structural and racial barriers that exist for all people with SUD persist through pregnancy and worsen postpartum in both access to and adequacy of treatment. Providers for the dyad can serve as another point in the healthcare system at which patients can be appropriately screened and adequately referred to treatment, and therefore should be aware of all resources available to aid in breaking the cycle (see Table 3). Although it is established that punitive substance use policies increase adverse perinatal outcomes (143, 144), the unjust and inhuman separation of the dyad is not widely acknowledged (145, 146). In short, care should be both evidence-based and person-centered, reflect science and public health but also grounded in human rights, human dignity, and recognize the unique burdens we place on pregnant people, their bodies, and their minds.

**Table 3:** Resources for providers.

Resources for providers		Application
Movement for Family Power	https://www.movementforfamilypower.org/	Advocacy, Legal, Policy
Drug Policy Alliance	https://drugpolicy.org/	Advocacy, Legal, Policy
Academy of Perinatal Harm Reduction	https://www.perinatalharmreduction.org/	Advocacy, Clinical
Center on Parenting and Opioids	https://cpo.uoregon.edu/sites/cpo2.uoregon.edu/files/2022-05/substance-use-and-recovery-in-pregnancy-and-early-parenting-full_0.pdf	Clinical. Policy
SAMHSA	https://store.samhsa.gov/sites/default/files/d7/priv/sma18-5054.pdf	Clinical, Legal
NNPQC	https://www.cdc.gov/reproductivehealth/maternalinfanthealth/nnpqc.htm	Clinical
National Clinical Consultation Center: Substance Use Warmline	Substance Use Warmline (855) 300 3595, https://nccc.ucsf.edu/clinical-resources/substance-use-resources/	Clinical
